# Joint association of vitamin D deficiency and sleep disorders with cardiovascular mortality: a prospective cohort study

**DOI:** 10.3389/fnut.2025.1514529

**Published:** 2025-04-11

**Authors:** Shizhen Wang, Dahong Zheng, Hui Wang, Mengru Wu, Wangjie Xia, Zhen Luo, Li Tian

**Affiliations:** ^1^The First Affiliated Hospital of Soochow University, Suzhou, China; ^2^School of Nursing, Medical College of Soochow University, Suzhou, China; ^3^Yangzhou Hospital of Traditional Chinese Medicine, Yangzhou, China

**Keywords:** joint association, vitamin D deficiency, sleep disorders, mortality, NHANES

## Abstract

**Purpose:**

Vitamin D deficiency and sleep disorders may independently contribute to increased mortality, but the combined effects of these two factors on mortality remain unknown. This study aimed to investigate both the separate and joint effects of vitamin D deficiency and sleep disorders on cardiovascular disease mortality, as well as all-cause mortality and cancer mortality.

**Methods:**

We analyzed data from 24,566 adults in the National Health and Nutrition Examination Survey (NHANES) from 2005 to 2018. Sleep disorders were assessed using self-report questionnaires, and vitamin D levels were measured through serum total 25-hydroxyvitamin D [25(OH)D]. Cox proportional hazards models were employed to evaluate the associations between separate and joint effects of vitamin D deficiency and sleep disorders with mortality outcomes.

**Results:**

Over a median follow-up of 9.08 years, we included a total of 24,566 individuals, in this study. Among them, 2,776 cases were all-cause deaths, 858 were cardiovascular disease deaths, and 644 were cancer deaths. We found that Vitamin D deficiency was independently associated with an increased risk of all-cause mortality, while sleep disorders were similarly associated with a higher risk of all-cause mortality. Notably, participants with both vitamin D deficiency and sleep disorders exhibited a significantly higher risk of all-cause mortality (HR, 2.31; 95% CI: 1.36–3.91) and cardiovascular mortality (HR, 2.39; 95% CI, 1.03–5.58) compared to those with only one or neither risk factor, even after adjusting for potential confounders.

**Conclusion:**

Our study highlights that the combination of vitamin D deficiency and sleep disorders was associated with an increased risk of all-cause and cardiovascular mortality in adults. These findings might help to refine dietary and lifestyle intervention strategies for this population.

## 1 Introduction

Cardiovascular diseases (CVDs) remain the leading cause of death worldwide, accounting for approximately 19.8 million fatalities in 2022 (1). The high prevalence and mortality associated with CVDs pose significant public health challenges, leading to severe consequences such as myocardial infarction, stroke, and heart failure. These conditions often result in long-term disability, diminished quality of life and impose a substantial financial burden on healthcare systems globally (2). As the global burden of CVDs intensifies, addressing modifiable risk factors is crucial for informing effective prevention strategies. Among these factors, improving poor dietary habits and lifestyle choices is essential in mitigating the risk of CVDs, as these modifiable elements, either independently or synergistically, play a pivotal role in disease development.

Vitamin D, a fat-soluble vitamin and steroid hormone, plays an indispensable role in sustaining bone health and mineral metabolism and also exerts extensive effects on the immune and nervous systems, including anti-inflammatory and immunomodulatory properties (3–5). Emerging evidence has linked vitamin D deficiency to various health conditions, including CVDs, diabetes, respiratory disorders, and cancers (6, 7). Despite extensive research, the relationship between vitamin D levels and mortality, whether from all causes, cardiovascular or cancer-specific, remains inconclusive. Although observational studies (8) suggest an association between low vitamin D levels and increased mortality risk, findings from randomized controlled trials (RCTs) have been inconsistent. Some meta-analyses of RCTs (9) have reported no significant reduction in all-cause or cardiovascular mortality with vitamin D supplementation. However, accumulating evidence, including findings from some RCTs and epidemiological studies, suggests a potential benefit in reducing cancer-specific mortality (9, 10) These discrepancies may stem from variations in study design, baseline vitamin D levels, and intervention strategies, necessitating further investigation into the complex relationship between vitamin D and mortality.

Sleep disorders, including insomnia and sleep apnea, are also recognized as significant health issues, with mounting evidence linking them to higher risks of cardiovascular disease, metabolic disorders, and overall mortality (11–16). While some studies have shown that sleep disorders are associated with an increased risk of cardiovascular events (17, 18), other research has observed differing associations across various populations (19, 20), emphasizing the inconsistency in findings and indicating the need for further investigation into the nature of this relationship.

While extensive research has explored the individual impacts of vitamin D deficiency and sleep disorders on health, there remains a glaring gap in the literature regarding their combined effects on adults mortality. This gap is particularly pressing as changes in sleep behavior often lead to altered eating habits, reduced outdoor activity, and decreased sunlight exposure, all of which can contribute to diminished vitamin D synthesis (21, 22). Furthermore, recent meta-analyses (23, 24) have highlighted a notable association between vitamin D deficiency and an increased susceptibility to sleep disorders and have suggested that taking vitamin D supplements may help improve sleep quality. Considering the intricate interplay between vitamin D levels and sleep health, a novel and comprehensive evaluation of their combined effects could offer valuable insights into their impact on cardiovascular disease outcomes, especially for those experiencing both significant sleep disorders and vitamin D deficiency, who are at elevated risk.

To address these gaps, our study aimed to investigate the prevalence of vitamin D deficiency and sleep disorders in a nationally representative cohort of U.S. adults and to evaluate both the individual and combined effects of these factors on cardiovascular, all-cause, and cancer-specific mortality. Utilizing comprehensive NHANES data from 2005 to 2018, this analysis sought to shed crucial light on the combined effects of these prevalent health conditions and their impact on mortality outcomes, thereby offering significant contributions to public health discourse. Moreover, the findings from this study were poised to establish a foundation for future research efforts focused on developing targeted interventions designed to mitigate the health risks associated with vitamin D deficiency and sleep disorders.

## 2 Methods

### 2.1 Data source and study population

**Figure 1:**
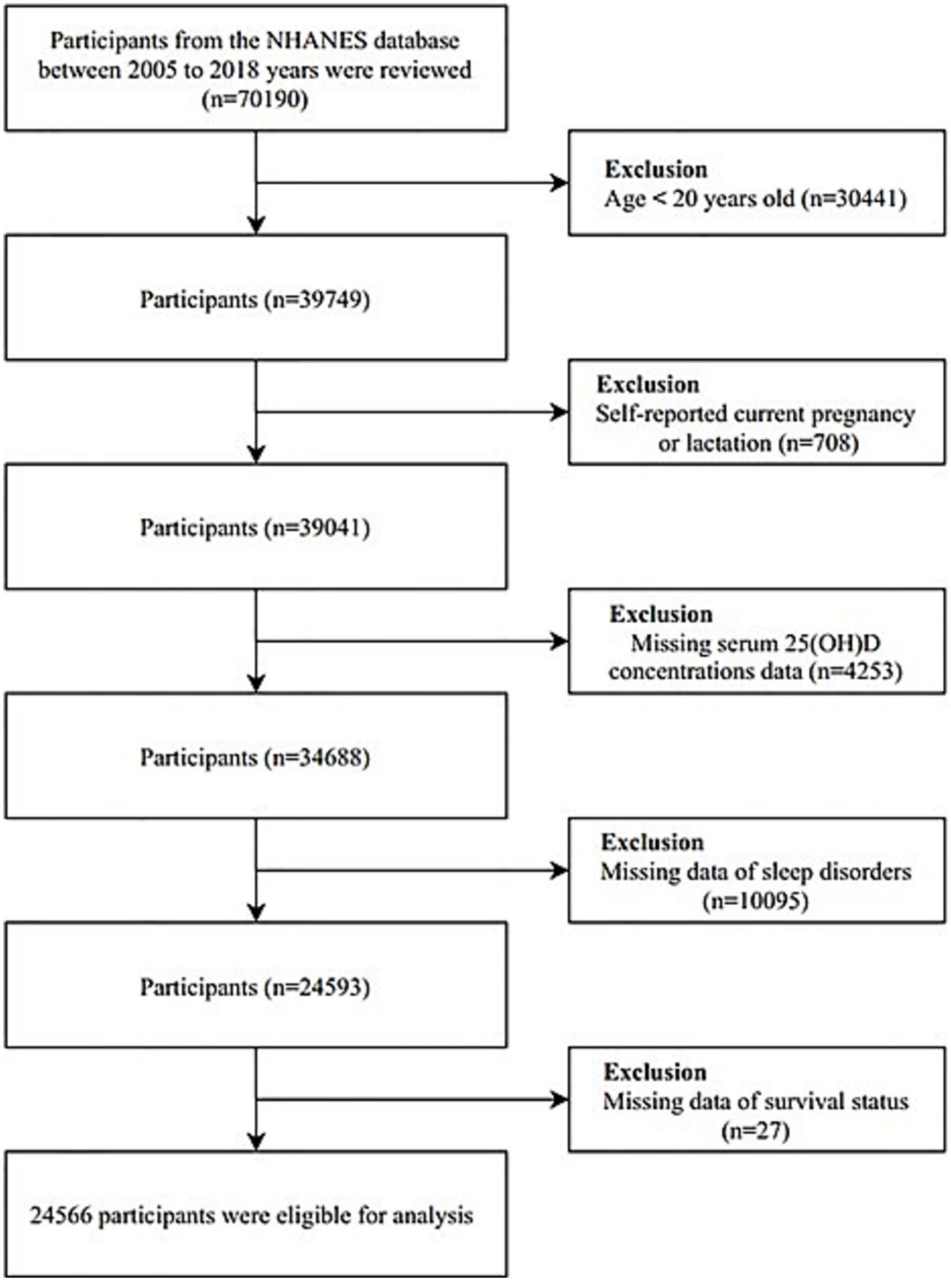
Flow chart of study participants.

The National Health and Nutrition Examination Survey (NHANES), a comprehensive program administered by the Centers for Disease Control and Prevention (CDC) and the National Center for Health Statistics (NCHS), provided the data for this study. NHANES is designed to assess the health and nutritional status of the U.S. population and adheres to the STROBE guidelines for reporting observational studies. The study protocol was approved by the NCHS Research Ethics Review Board, and written informed consent was obtained from all participants. Data for this analysis were drawn from six consecutive NHANES cycles covering 2005 to 2018. The dataset comprised demographic information, health conditions, physical examination results, and responses to questionnaires. In the initial phase of our analysis, we excluded 30,441 individuals who were under the age of 20 and 708 participants who self-reported as pregnant or lactating. Additional exclusions were made for participants with incomplete data on sleep disorders, serum 25(OH)D levels, and survival status during the follow-up period. The detailed participant selection process is illustrated in Figure 1.

### 2.2 Measurement of vitamin D and sleep disorders

Vitamin D levels were evaluated from a single serum sample collected at baseline from NHANES participants. These samples were processed and stored securely at −30°C before being sent to the Nutritional Biomarkers Branch at the Division of Laboratory Sciences, National Center for Environmental Health. The analysis focused on total serum 25-hydroxyvitamin D [25(OH)D], which includes both 25(OH)D2 and 25(OH)D3. The concentration of 25(OH)D was determined using high-performance liquid chromatography–tandem mass spectrometry (HPLC–MS/MS) technique, as standardized by the CDC. Methodological specifics are documented and accessible on the NHANES website. Serum 25(OH)D levels were categorized based on established cut-off points: (1) Vitamin D deficiency, defined as serum 25(OH)D concentrations <20 ng/mL (< 50 nmol/L), and (2) Vitamin D non-deficiency, defined as serum 25(OH)D concentrations ≥20 ng/mL (≥ 50 nmol/L) (7, 25).

Sleep disorders were assessed using the Sleep Disorders Questionnaire, where individuals were classified as having a sleep disorder if they responded affirmatively to the question, “Have you been diagnosed with a sleep disorder?” (26) Based on their responses, participants were categorized into “yes” or “no” groups. Individuals who did not provide a response or were unsure about their condition were designated as having missing data.

### 2.3 Ascertainment of mortality

The primary outcomes of this study were all-cause mortality, cardiovascular mortality, and cancer-specific mortality. Mortality status was determined using the NHANES public-use linked mortality file, updated through December 31, 2019, and linked to the National Death Index (NDI) via a probabilistic matching algorithm. The underlying causes of death were classified based on the International Statistical Classification of Diseases and Related Health Problems, Tenth Revision (ICD-10). This study specifically analyzed mortality from all causes: cancer (codes C00–C97), CVD (codes I00–I09, I11, I13, and I20–I51), and other causes.

### 2.4 Covariates

Potential covariates were selected based on previous literature and prior knowledge regarding the relationship between lifestyle factors and mortality. In the study, specific covariates were analyzed for both descriptive and inferential statistics. (i) Sociodemographic characteristics included age, sex (male or female), race/ethnicity (non-Hispanic White, non-Hispanic Black, Mexican American or Hispanic, and others), family income-to-poverty ratio (<1.3, 1.3–3.49, and ≥ 3.5), educational level (less than high school, high school, and college or higher), and health insurance status (no insurance, government insurance, and private insurance). (ii) Behavioral factors assessed in the study included smoking status (never, former, and current), alcohol consumption (yes, no), systemic immune-inflammation index (SII), which was calculated as platelet count × neutrophil count / lymphocyte count (27), physical activity, and BMI. (iii) Health-related factors were based on self-reported physician diagnoses of hypertension, diabetes mellitus, hyperlipidemia, CVD, and cancer. A detailed definition of the covariates can be found in Supplementary Table S1.

### 2.5 Statistical analysis

All analyses were adjusted for the complex sampling design of NHANES, including sample weights, clustering, and stratification, to ensure accurate data analysis. Baseline characteristics were stratified by sleep disorder status (no sleep disorder vs. sleep disorder). Continuous variables were presented as survey-weighted means with 95% confidence intervals (CI), while categorical variables were reported as percentages with corresponding 95% CI. Cox proportional hazards regression models were applied to explore the associations between vitamin D status, sleep disorders, and mortality outcomes (cardiovascular, all-cause, and cancer mortality). To obtain more accurate estimates and to examine the detailed patterns of the relationship between serum vitamin D levels and mortality, we employed restricted cubic spline models with knots positioned at the 5th, 35th, 65th, and 95th percentiles (28).

The analysis was structured into three models: Model 1 was implemented as a crude model without adjustments. Model 2 adjusted for age, sex, race/ethnicity, educational attainment, poverty-income ratio (PIR), and health insurance status. The fully adjusted model 3 included additional covariates such as smoking, alcohol consumption, BMI, physical activity, SII, and a history of hypertension, diabetes mellitus (DM), hyperlipidemia, CVD, and cancer. Interaction analyses were performed to assess whether the combined effects of vitamin D status and sleep disorders on mortality outcomes exceeded their individual effects on additive and multiplicative scales. For additive interaction, the relative excess risk due to interaction (RERI) was calculated following the methodology proposed by Knol and VanderWeele (29). The ratio of hazard ratios (RHR) was calculated for multiplicative interaction. Subgroup analyses were conducted based on age, sex, and other relevant variables to explore potential association variations. Sensitivity analyses were also conducted by excluding participants with less than 3 years of follow-up to assess the robustness of the findings.

All statistical analyses were conducted using SAS version 9.4 (SAS Institute, Cary, NC), employing survey-specific modules (surveymeans, surveyfreq, surveyphreg) to account for the NHANES complex sampling design. A two-sided *p* value <0.05 was considered statistically significant.

## 3 Results

### 3.1 Baseline characteristics

Supplementary Table S2 presents the baseline characteristics of the study population stratified by sleep disorder status. Among the 24,566 participants, the mean age was 49.8 years (standard error [SE], 17.8), and 51.9% were male. The mean age was 49.4 years (SE, 18.0) for those without sleep disorders and 53.7 years (SE, 15.3) for those with sleep disorders. The median serum vitamin D concentration was 59.9 nmol/L (interquartile range [IQR], 43.5 to 77.4), and the median body mass index (BMI) was 27.9 kg/m^2^ (IQR, 24.3 to 32.4). Participants with sleep disorders were significantly more likely to be male (*p* = 0.004), non-Hispanic White (*p* < 0.001), have a lower PIR (*p* < 0.001), lower educational attainment (*p* = 0.001), and private health insurance (*p* < 0.001). They were also more likely to have a history of smoking (*p* = 0.02), hypertension (*p* < 0.001), and hyperlipidemia (*p* < 0.001), but less likely to have a history of diabetes (*p* < 0.001), cardiovascular disease (*p* < 0.001), or cancer (*p* < 0.001). Additionally, the sleep disorder group had significantly higher SII (*p* = 0.01) and lower physical activity levels (*p* < 0.001). No significant difference in vitamin D levels was observed between the groups (*p* = 0.24, see Supplementary Table S2).

### 3.2 Relationship between vitamin D deficiency, sleep disorders and mortality

In this cohort of 24,566 participants, we analyzed the association between sleep disorder status, vitamin D deficiency status, and mortality separately. Among individuals with sleep disorders, 318 died from all causes, 92 from cardiovascular disease (CVD), and 63 from cancer. Among those with vitamin D deficiency, 935 died from all causes, 273 from CVD, and 221 from cancer. Table 1 presents the associations between vitamin D deficiency, sleep disorders, and mortality outcomes, covering all-cause, cardiovascular, and cancer mortality. After adjusting for relevant covariates, vitamin D deficiency was significantly associated with an elevated risk of all-cause mortality (HR: 1.40, 95% CI: 1.11–1.77). However, the associations between vitamin D deficiency and cardiovascular mortality (HR: 1.39, 95% CI: 0.92–2.10) and cancer mortality (HR: 1.49, 95% CI: 0.93–2.38) did not reach statistical significance. Similarly, participants with sleep disorders had a significantly higher risk of all-cause mortality (HR: 1.35, 95% CI: 1.00–1.81) compared to those without sleep disorders. Nevertheless, sleep disorders were not significantly associated with increased risks of cardiovascular mortality (HR: 1.45, 95% CI: 0.93–2.29) or cancer-specific mortality (HR: 1.03, 95% CI: 0.53–1.99).

**Table 1:** Association of sleep disorders and vitamin D deficiency with all-cause, cardiovascular disease and cancer mortality.

Mortality	Sleep disorders HR (95% CI)		Vit D deficiency HR (95% CI)		*P*-interaction
Outcome	No	Yes	No	Yes	
All-cause mortality	2458/18774	318/1774	1841/13720	935/6828	0.16
Model 1^a^	ref	1.60 (1.34–1.91)	ref	1.21 (1.05–1.39)	
Model 2^b^	ref	1.51 (1.26–1.80)	ref	1.55 (1.36–1.77)	
Model 3^c^	ref	1.35 (1.00–1.81)	ref	1.40 (1.11–1.77)	
CVD mortality	766/17082	92/1548	585/12464	273/6166	0.52
Model 1^a^	ref	1.70 (1.28–2.26)	ref	1.17 (0.92–1.47)	
Model 2^b^	ref	1.73 (1.28–2.33)	ref	1.60 (1.28–2.01)	
Model 3^c^	ref	1.45 (0.93–2.29)	ref	1.39 (0.92–2.10)	
Cancer mortality	581/16897	63/1519	423/12302	221/6114	0.46
Model 1^a^	ref	1.11 (0.78–1.56)	ref	1.12 (0.87–1.45)	
Model 2^b^	ref	0.98 (0.66–1.44)	ref	1.63 (1.22–2.17)	
Model 3^c^	ref	1.03 (0.53–1.99)	ref	1.49 (0.93–2.38)	

Multivariate weighted Cox proportional hazards regression models were used, and 24,566 adults from NHANES were eligible for the present analysis.

^aUnadjusted logistic regression model.^

^bAdjusted for age, sex, race/ethnicity, educational status, PIR and health insurance.^

^cAdjusted for Model 2 and further adjusted for smoking, alcohol consumption, BMI, physical activity, SII and history of CVD, DM, hyperlipidemia, hypertension and cancer.^

HR, hazard ratio; CI, confidence interval; NHANES, National Health and Nutrition Examination Survey; PIR, poverty-to-income ratio; BMI, body mass index; SII, Systemic Immune-Inflammation Index; CVD, cardiovascular disease; DM, diabetes mellitus.

### 3.3 Dose–response relationship of serum total 25-hydroxyvitamin D (25(OH)D) levels and mortality

**Figure 2:**
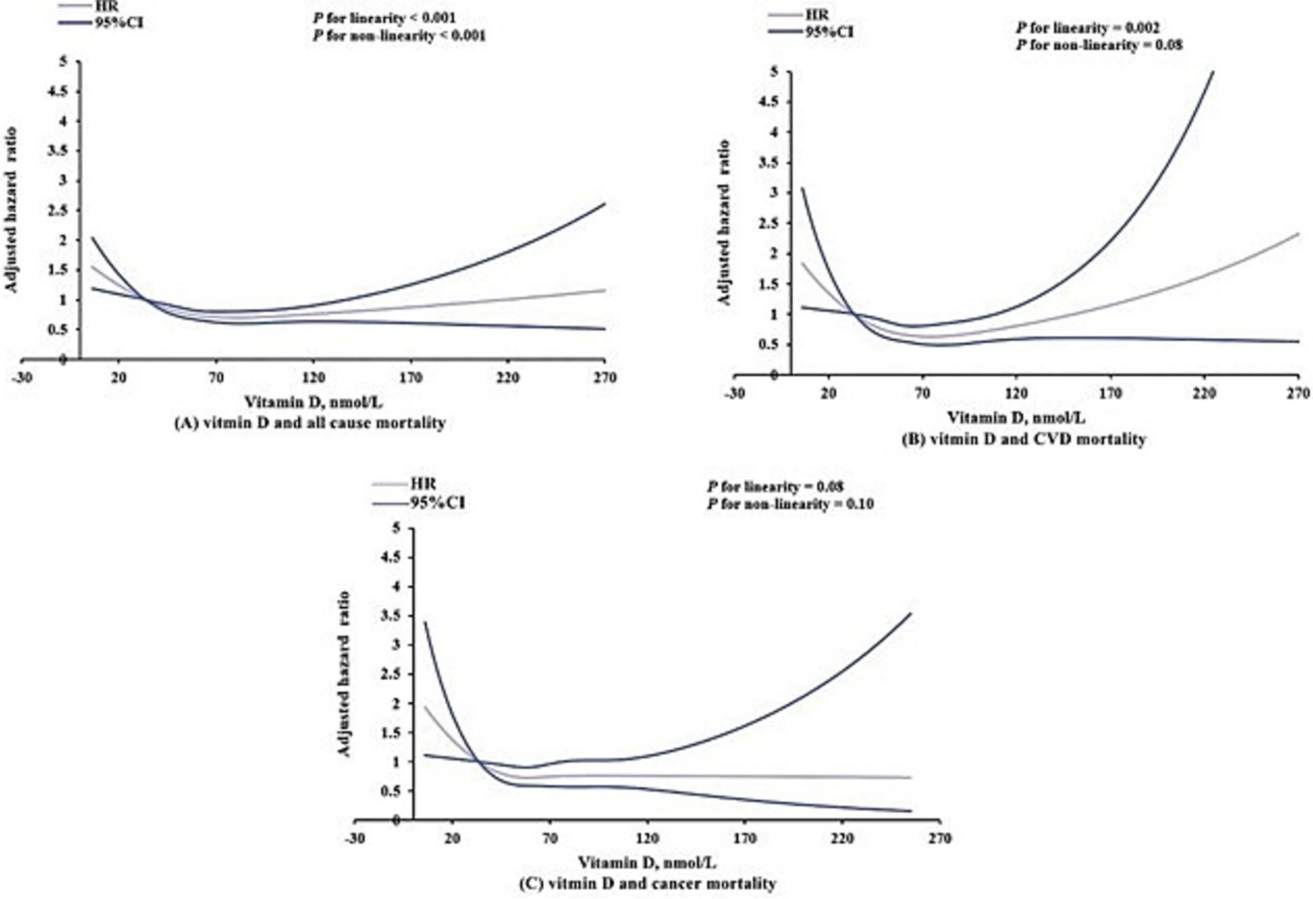
Dose–response association of serum vitamin D levels with all-cause **(A)**, cardiovascular **(B)**, and cancer **(C)** mortality among US adults aged 20 years or older. Models were adjusted for age, sex, race/ethnicity, educational status, poverty-to-income ratio, health insurance, smoking, alcohol consumption, body mass index, physical activity, Systemic Immune-Inflammation Index and history of cardiovascular disease, diabetes mellitus, hyperlipidemia, hypertension and cancer.

Further analysis with restricted cubic splines (Figure 2) revealed varying relationships between serum vitamin D levels and different mortality outcomes. Specifically, a significant J-shaped relationship was observed between vitamin D levels and all-cause mortality (linear *p*-value <0.001; nonlinear *p*-value <0.001), indicating that both deficient and excessive vitamin D levels were associated with increased risk of death. Regarding cardiovascular mortality, a significant linear relationship was identified (*p* = 0.002). Although a J-shaped trend was observed, the nonlinear *p*-value of 0.08 did not reach statistical significance, suggesting that the relationship between vitamin D levels and cardiovascular mortality is statistically more consistent with a linear model. Conversely, no statistically significant linear (*p* = 0.08) or nonlinear (*p* = 0.10) associations were found between vitamin D levels and cancer mortality (Figure 2).

**Figure 3:**
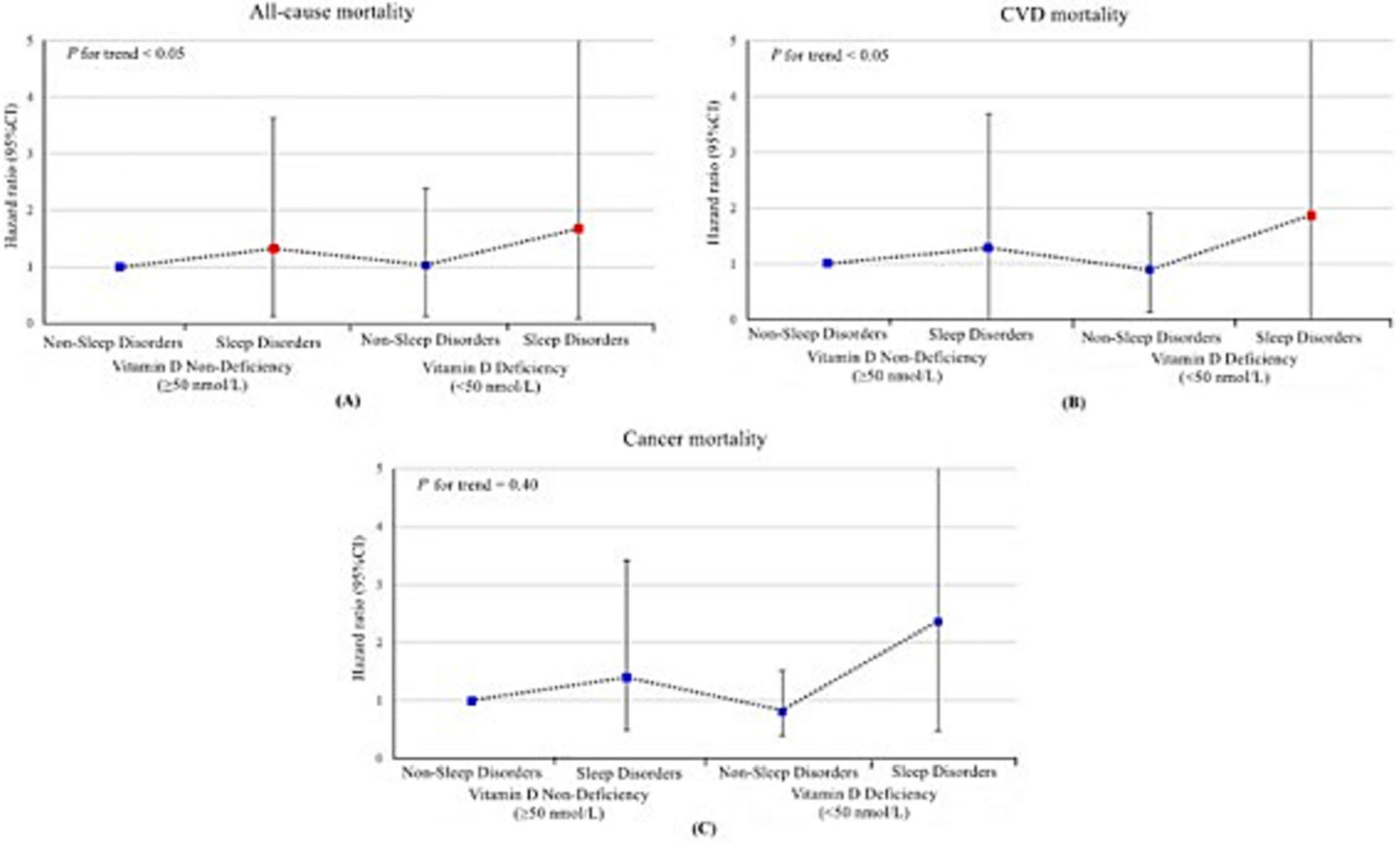
Joint association of sleep disorders and vitamin D deficiency with all-cause **(A)**, cardiovascular disease **(B)** and cancer **(C)** mortality among US adults aged 20 years or older, National Health and Nutrition Examination Survey 2005–2018. The hazard ratios and their respective 95% confidence interval are represented by a solid symbols and error bars. Red markers indicate statistically significant associations (*p* < 0.05), while blue markers indicate non-significant associations. Models were adjusted for age, sex, race/ethnicity, educational status, poverty-to-income ratio, health insurance, smoking, alcohol consumption, body mass index, physical activity, Systemic Immune-Inflammation Index and history of cardiovascular disease, diabetes mellitus, hyperlipidemia, hypertension and cancer. HR, hazard ratio; CI, confidence interval; CVD, cardiovascular disease.

### 3.4 Joint association of vitamin D deficiency and sleep disorders with mortality

In the joint analyses, individuals with both vitamin D deficiency and sleep disorders demonstrated the highest risks of all-cause mortality, cardiovascular mortality, and cancer mortality. Specifically, the hazard ratios (HRs) for all-cause mortality and cardiovascular mortality were significantly elevated at 2.31 (95% CI, 1.36–3.91) and 2.39 (95% CI, 1.03–5.58), respectively, compared to other groups in the fully adjusted model. In contrast, the association between these combined risk factors and cancer mortality was not statistically significant, with an HR of 2.02 (95% CI, 0.68–5.97) (Table 2 and Figure 3). However, no statistically significant interaction between vitamin D deficiency and sleep disorders was observed, either on the additive or multiplicative scale, which indicated that while the combined presence of these two risk factors was associated with an elevated risk of mortality, their interaction did not significantly amplify this risk beyond what would be expected from their individual effects (Supplementary Table S3). Sensitivity analysis further validated the robustness of these associations, as the results remained consistent after excluding patients with follow-up durations of less than 3 years (Table 3).

**Table 2:** Joint association of sleep disorders and vitamin D deficiency with all-cause, cardiovascular and cancer mortality.

Mortality outcome	No vitamin D deficiency, no sleep disorders HR (95% CI)	No vitamin D deficiency, sleep disorders HR (95% CI)	Vitamin D deficiency, no sleep disorders HR (95% CI)	Vitamin D deficiency and sleep disorders HR (95% CI)	*P*-trend
All-cause mortality	1644/12527	814/6247	197/1193	121/581	
Model 1^a^	ref	1.17 (1.02–1.35)	1.50 (1.22–1.83)	2.14 (1.54–2.97)	<0.001
Model 2^b^	ref	1.52 (1.32–1.74)	1.43 (1.18–1.72)	2.46 (1.77–3.41)	<0.001
Model 3^c^	ref	1.32 (1.03–1.68)	1.20 (0.91–1.60)	2.31 (1.36–3.91)	0.002
CVD mortality	519/11402	247/5680	66/1062	26/486	
Model 1^a^	ref	1.14 (0.91–1.42)	1.61 (1.19–2.19)	2.16 (1.13–4.12)	0.001
Model 2^b^	ref	1.58 (1.27–1.96)	1.68 (1.22–2.30)	2.83 (1.48–5.41)	<0.001
Model 3^c^	ref	1.29 (0.89–1.88)	1.31 (0.76–2.26)	2.39 (1.03–5.58)	0.02
Cancer mortality	383/11266	198/5631	40/1036	23/483	
Model 1^a^	ref	1.10 (0.84–1.45)	1.06 (0.68–1.64)	1.36 (0.68–2.69)	0.35
Model 2^b^	ref	1.60 (1.16–2.20)	0.89 (0.54–1.48)	1.80 (0.93–3.48)	0.049
Model 3^c^	ref	1.40 (0.83–2.38)	0.92 (0.44–1.91)	2.02 (0.68–5.97)	0.40

Multivariate weighted Cox proportional hazards regression models were used, and 24,566 adults from NHANES were eligible for the present analysis.

^aUnadjusted logistic regression model.^

^bAdjusted for age, sex, race/ethnicity, educational status, PIR and health insurance.^

^cAdjusted adjusted for Model 2 and further adjusted for smoking, alcohol consumption, BMI, physical activity, SII and history of CVD, DM, hyperlipidemia, hypertension and cancer.^

HR, hazard ratio; CI, confidence interval; NHANES, National Health and Nutrition Examination Survey; PIR, poverty-to-income ratio; BMI, body mass index; SII, Systemic Immune-Inflammation Index; CVD, cardiovascular disease; DM, diabetes mellitus.

### 3.5 Subgroup analysis

Supplementary Figure S1 illustrates the subgroup analysis of the combined effects of vitamin D deficiency and sleep disorders on all-cause mortality, cardiovascular mortality, and cancer mortality. The joint effect was significant across most subgroups for all-cause mortality, especially among individuals without a history of diabetes, CVD, or cancer. For cardiovascular mortality, the combined effect remained significant across various subgroups, particularly among individuals without hyperlipidemia or CVD. No significant joint effect was observed for cancer mortality, although income level and BMI showed significant interactions in some subgroups. Notably, individuals with a BMI ≥ 25 had a lower risk of cancer mortality. In summary, the combined effects of vitamin D deficiency and sleep disorders on all-cause and cardiovascular mortality were consistent across various demographic and health-related subgroups. However, the impact on cancer mortality did not show a consistent pattern across subgroups (Supplementary Figure S1).

**Table 3:** Joint association of sleep disorders and vitamin D deficiency with all-cause, cardiovascular disease and cancer mortality (excluding follow-ups shorter than 3 years).

Mortality outcome	No vitamin D deficiency, no sleep disorders HR (95% CI)	No vitamin D deficiency, sleep disorders HR (95% CI)	Vitamin D deficiency, no sleep disorders HR (95% CI)	Vitamin D deficiency and sleep disorders HR (95% CI)	*P-*trend
All-cause mortality	1269/12152	614/6047	144/1140	85/545	
Model 1^a^	ref	1.15 (1.00–1.33)	1.45 (1.12–1.87)	2.13 (1.42–3.19)	<0.001
Model 2^b^	ref	1.48 (1.27–1.71)	1.40 (1.09–1.79)	2.39 (1.57–3.65)	<0.001
Model 3^c^	ref	1.19 (0.91–1.54)	1.20 (0.82–1.75)	2.46 (1.29–4.72)	0.02
CVD mortality	401/11284	180/5613	45/1041	21/481	
Model 1^a^	ref	1.04 (0.80–1.36)	1.37 (0.87–2.16)	2.47 (1.18–5.15)	0.03
Model 2^b^	ref	1.42 (1.09–1.85)	1.49 (0.96–2.32)	3.16 (1.47–6.78)	0.001
Model 3^c^	ref	1.06 (0.65–1.72)	1.14 (0.63–2.07)	3.45 (1.44–8.24)	0.046
Cancer mortality	292/11175	152/5585	30/1026	16/476	
Model 1^a^	ref	1.18 (0.90–1.55)	1.05 (0.68–1.62)	1.36 (0.58–3.17)	0.27
Model 2^b^	ref	1.66 (1.19–2.30)	0.85 (0.51–1.43)	1.77 (0.78–4.04)	0.052
Model 3^c^	ref	1.57 (0.93–2.64)	1.14 (0.57–2.25)	2.66 (0.88–8.08)	0.10

Multivariate weighted Cox proportional hazards regression models were used, and 24,566 adults from NHANES were eligible for the present analysis.

^aUnadjusted logistic regression model.^

^bAdjusted for age, sex, race/ethnicity, educational status, PIR and health insurance.^

^cAdjusted for Model 2 and further adjusted for smoking, alcohol consumption, BMI, total physical activity, SII and history of CVD, DM, hyperlipidemia, hypertension and cancer.^

HR, hazard ratio; CI, confidence interval; NHANES, National Health and Nutrition Examination Survey; PIR, poverty-to-income ratio; BMI, body mass index; SII, Systemic Immune-Inflammation Index; CVD, cardiovascular disease; DM, diabetes mellitus.

## 4 Discussion

This study explored the combined effect of vitamin D deficiency and sleep disorders on mortality risks using a nationally representative cohort of 24,566 U.S. adults. Approximately 34.34% of participants were found to have vitamin D deficiency, while 8.41% had sleep disorders. Over a median follow-up of 9.08 years, both vitamin D deficiency and sleep disorders were individually associated with significantly increased risks of all-cause mortality. However, neither condition alone was significantly associated with cardiovascular or cancer mortality. In the joint analysis, individuals with both conditions exhibited markedly higher risks of all-cause and cardiovascular mortality compared to those with only one or neither risk factor. The combined effect on cancer mortality was not statistically significant. These associations remained robust in sensitivity and subgroup analyses. To the best of our knowledge, this is the first study to investigate the joint effects of vitamin D deficiency and sleep disorders on mortality risks in U.S. adults, underscoring the need to address both factors to mitigate all-cause and cardiovascular mortality in this population.

Growing evidence suggests that the relationship between vitamin D levels and cardiovascular mortality is not uniform, with varying dose–response patterns reported across studies. Our analysis, based on six consecutive NHANES cycles (2005–2018) in adults aged 20 years and older, identified a linear association between vitamin D levels and cardiovascular mortality, indicating that lower vitamin D levels are consistently linked to higher cardiovascular mortality risk. This finding aligns with Mo et al. (30) who similarly reported a linear relationship between serum 25-hydroxyvitamin D concentrations and cardiovascular mortality risk. However, other studies have reported a non-linear association. Notably, a recent NHANES-based analysis spanning a broader timeframe (2001–2018) identified an L-shaped association, suggesting a threshold effect where cardiovascular mortality risk declines primarily at lower vitamin D levels before reaching a plateau (31). These discrepancies may arise from variations in study periods, population characteristics, analytical methodologies, or vitamin D assay standardization. Collectively, these findings underscore the complexity of vitamin D’s role in cardiovascular mortality and highlight the need for further research to refine supplementation strategies, clarify optimal vitamin D thresholds, and delineate the mechanisms underlying these associations.

Building on these findings, our study further supports the notion that both vitamin D deficiency and excessive serum 25(OH)D levels may contribute to increased mortality risk, reinforcing a J-shaped dose–response pattern for all-cause mortality. This pattern has been demonstrated in the CopD study, which reported an increased mortality risk when serum 25(OH)D levels exceeded 50–60 ng/mL (32). Such nonlinearity suggests that while maintaining adequate vitamin D levels is crucial, indiscriminate supplementation may not always yield additional benefits. Recent study recommend a daily vitamin D intake of up to 4,000 IU for normal-weight individuals and 10,000 IU for those with obesity to maintain serum 25(OH)D levels within 30–50 ng/mL (33). While our study suggests potential risks associated with excessive vitamin D levels, prior research indicates that commonly used high-dose supplementation (e.g., 50,000 IU/week) does not necessarily lead to toxicity (34). Despite strong observational evidence linking low vitamin D levels to increased mortality risk (8), RCTs have produced inconsistent findings, particularly regarding all-cause and cardiovascular mortality. Some RCTs (35) have indicated a reduction in all-cause mortality, while others (9, 36) reported no significant impact on all-cause or cardiovascular mortality, though reductions in cancer mortality were observed. These discrepancies may arise from several methodological limitations in RCTs, such as suboptimal trial designs (37, 38), failure to selectively enroll individuals with severe vitamin D deficiency (e.g., <18 ng/mL) which may dilute the potential benefits of supplementation (39), and substantial heterogeneity in baseline vitamin D status, supplementation dosages, and follow-up durations across trials, making it challenging to detect consistent effects (40). Additionally, another critical but often overlooked factor is sunlight exposure, a key determinant of endogenous vitamin D synthesis. Inadequate sun exposure has been linked to increased mortality risk (41), yet many RCTs do not account for individual differences in UVB exposure, potentially leading to misclassification of vitamin D status and underestimation of supplementation benefits. These inconsistencies highlight the need for more targeted RCTs with improved methodological rigor. Optimized trial designs, such as those employed by Rostami et al. (42) and Dawson-Hughes et al. (43), which focus on high-risk populations with severe vitamin D deficiency and tailored intervention strategies, may provide more definitive evidence on the role of vitamin D in mortality outcomes. Additionally, emerging evidence suggests that vitamin D supplementation may be more beneficial when combined with vitamin K intake in managing cardiovascular risk factors (44). However, our study did not assess the role of vitamin K co-supplementation, highlighting the need for future research in this area. Future studies should prioritize these approaches to clarify the potential benefits of vitamin D supplementation in reducing mortality risk.

Regarding sleep disorders, our study found a significant association with all-cause mortality, consistent with prior research (15, 45). However, we did not observe statistically significant associations with cardiovascular or cancer-specific mortality, contrasting with reports from some studies (12, 46, 47) that identified such associations. These inconsistencies across studies may be attributed to the complex nature of sleep as a concept, differences in study populations, geographic variations in daily light exposure, limited statistical power in smaller studies, or potential biases in measurement techniques. Mechanistically, sleep disorders are known to promote systemic inflammation, elevating levels of C-reactive protein and interleukin-6, which are associated with increased mortality risk (45, 48). Moreover, sleep disorders may adversely affect health outcomes by influencing lifestyle factors such as physical activity, smoking, and alcohol consumption. For instance, prolonged sleep duration might reduce the time available for engaging in health-promoting activities, like physical activity (49, 50). In this study, we observed significantly lower physical activity levels in the sleep disorder group, further supporting this hypothesis. Overall, our findings reinforce the role of sleep disorders as a significant health risk factor, highlighting the need for heightened public awareness and targeted interventions to improve sleep health.

A systematic comparison of joint and interaction analyses offers a more nuanced understanding of how these two risk factors jointly influence mortality. We found that the combined effect of vitamin D deficiency and sleep disorders significantly increased both all-cause and cardiovascular mortality, and these associations remained robust across various subgroups. Furthermore, our findings persisted even after excluding participants with follow-up periods of less than 3 years, reinforcing the reliability of the results. This evidence offers valuable epidemiological insights for guiding public health policies and clinical interventions, emphasizing the need to optimize vitamin D levels and improve sleep quality to reduce early mortality risk associated with these modifiable factors. Although our additive and multiplicative interaction analyses did not reach statistical significance, this outcome suggests that capturing these complex interactions may require more extensive studies or more sensitive statistical methods. It also highlights the importance of not only focusing on the independent effects of single risk factors but also delving into the joint management of multiple risk factors. A review of randomized controlled studies has emphasized that such strategies, including nutrition counseling, dietary changes, addressing sedentary behavior, promoting physical activity, avoiding tobacco exposure, and minimizing long-term glucocorticoid therapy, could effectively manage multiple health risks (51). Overall, these findings underscore the necessity of integrating multiple health factors into comprehensive management strategies within clinical and public health practices, providing new avenues for enhancing population health and extending life expectancy.

Subgroup analyses revealed that the combined effects of vitamin D deficiency and sleep disorders on cardiovascular mortality remained consistently significant across various subgroups, particularly among individuals without hyperlipidemia or cardiovascular disease. Notably, the joint effects were more pronounced in individuals with a normal BMI, which aligns with prior research (52), indicating that the cumulative impact of vitamin D deficiency on cardiovascular mortality is most evident in normal-weight individuals. This heightened sensitivity may be attributed to the more evident protective cardiovascular effects of vitamin D in individuals with a normal BMI, such as improved endothelial function and blood pressure regulation (53). Conversely, the metabolic disruptions associated with obesity, including chronic inflammation and insulin resistance, could attenuate the impact of vitamin D deficiency on cardiovascular outcomes (54). Regarding cancer mortality, although the joint effects of vitamin D deficiency and sleep disorders were not statistically significant in our analysis, accumulating evidence suggests that BMI may modify the relationship between vitamin D and cancer outcomes. The VITAL study demonstrated that vitamin D supplementation significantly reduced the incidence of cancer in individuals with BMI <25 but not in those with higher BMI (55), supporting the hypothesis that vitamin D’s protective effects are more pronounced in individuals with lower BMI. A secondary analysis further revealed that vitamin D supplementation was associated with a reduced risk of advanced cancer, particularly among participants with BMI <25 (56). The attenuated effect of vitamin D in individuals with higher BMI may be explained by differences in vitamin D distribution. While previous studies suggested that sequestration of vitamin D in adipose tissue reduces its bioavailability (57), emerging evidence indicates that volumetric dilution—whereby the larger distribution volume in obese individuals results in lower circulating vitamin D concentrations—may be a more plausible mechanism (58). This dilution effect likely reduces the physiological availability of vitamin D, potentially contributing to the weaker association between vitamin D status and cancer mortality observed in individuals with obesity. Additionally, recent findings by Muñoz and Grant (10) reinforce the role of vitamin D in reducing cancer mortality, emphasizing the importance of maintaining adequate vitamin D levels, particularly among individuals with lower BMI. Collectively, these findings underscore the need for further research to elucidate BMI’s role as a key modifier in the relationship between vitamin D and cancer outcomes, which may inform future precision-based supplementation strategies.

Several biological pathways may account for the observed joint effects of vitamin D deficiency and sleep disorders on all-cause and cardiovascular mortality, necessitating further exploration. Firstly, vitamin D plays a critical role in regulating circadian rhythms and maintaining sleep quality, particularly in brain regions like the hypothalamus, where its receptors are abundantly expressed (59, 60). Deficiency in vitamin D may disrupt the function of these brain regions and lower melatonin levels, potentially leading to sleep disorders and consequently increasing risks of all-cause and cardiovascular mortality (22, 61). Moreover, vitamin D deficiency is associated with immune system dysregulation and chronic inflammatory responses, promoting elevated levels of inflammatory mediators such as TNF-*α*, IL-1, and prostaglandin D2, which are closely linked to sleep regulation (62, 63). Furthermore, vitamin D deficiency can alter gut microbiota composition, potentially disrupting B vitamin metabolism and reducing pantothenic acid production, which is essential for immune regulation (64). This imbalance may impair immune function and promote a pro-inflammatory state associated with atherosclerosis and autoimmunity (65). Given the strong link between chronic inflammation and cardiovascular disease, these immune disturbances may further heighten cardiovascular risks in individuals with both vitamin D deficiency and sleep disorders (66). Additionally, disruptions in the gut microbiota—closely linked to immune and cardiovascular health—may further compound the risk of cardiovascular mortality in individuals with vitamin D deficiency and sleep disorders (67). However, within the complex pathology of cancer, these mechanisms might be overshadowed by other factors such as genetic predisposition, tumor type, disease stage, and treatment modalities (68, 69), making it challenging for current study designs to capture these complex interactions. Additionally, individual variability and the heterogeneity of cancer types may further complicate the impact of vitamin D deficiency and sleep disorders on cancer mortality, particularly among patients with different biological characteristics. This variability could contribute to the absence of significant joint or interactive effects observed in our overall analysis. A limitation of this study is the presence of unmeasured variables that may influence both vitamin D levels and sleep disorders, such as long-term medication use or psychosocial factors, which could potentially confound the results. Therefore, future research should aim for more comprehensive control of confounding factors and consider employing more advanced statistical and bioinformatics methods to gain a deeper understanding of the complex effects of vitamin D deficiency and sleep disorders on cancer mortality.

This study possesses several notable strengths. It is the first to examine the combined effects of vitamin D deficiency and sleep disorders on mortality, addressing a significant gap in existing literature. Using a large, nationally representative sample from NHANES, alongside the prospective design and extended follow-up period, enhances the robustness and generalizability of our findings. Additionally, extensive adjustments for potential confounders in sensitivity and subgroup analyses further strengthen the validity of our results, providing a comprehensive understanding of these associations. However, several limitations should be acknowledged. First, all exposures and confounders were measured at baseline, and any changes during the follow-up period were not captured, which could affect the observed associations. Second, our exposure variables relied primarily on self-reported data, which may be subject to recall bias and random measurement errors, potentially leading to underestimating the true magnitude of the associations. Moreover, although sleep disorders were assessed by physicians, the absence of detailed reporting on the specific nature hindered further in-depth analysis, limiting our ability to explore the multidimensional nature of sleep disturbances. Third, despite careful consideration of multiple confounding factors, the observational nature of the study design means that residual confounding and reverse causality cannot be entirely ruled out. We attempted to mitigate the impact of reverse causation by excluding patients who died within the first 3 years of follow-up in our sensitivity analysis, yet this limitation warrants caution. Finally, although the NHANES sample was substantial, it may not fully represent the U.S. population, as the participants were self-selected, limiting our findings’ generalizability to other regions of the U.S. and to other countries.

## 5 Conclusion

In summary, this population-based cohort study underscores the significant joint effect of vitamin D deficiency and sleep disorders on all-cause and cardiovascular mortality risk. The implications of this research are promising, as addressing both vitamin D status and sleep health may offer a more comprehensive approach to reducing mortality risk in clinical practice and public health interventions. The potential for future research to build on our findings, focusing on longitudinal studies with repeated measurements and personalized supplementation strategies, offers hope for further advancements in this field.

## Data Availability

The original contributions presented in the study are included in the article/Supplementary material, further inquiries can be directed to the corresponding authors.
