# Supplementary material for: Joint association of vitamin D deficiency and sleep disorders with cardiovascular mortality: a prospective cohort study
Source: Front Nutr. 2025 Apr 11;12:1514529. doi: 10.3389/fnut.2025.1514529 (PMC12021624; doi:10.3389/fnut.2025.1514529)
Supplement: Supplementary file 1 [file Data_Sheet_1.docx]

**Supplementary materials**

**Table S1** The resource and definition of the selected covariates.

**Table S2** Baseline characteristics of the study participants by categories of sleep disorders.

**Table S3** Interaction test for the relationship of sleep disorders and vitamin D deficiency with mortality.

**Fig. S1** Forest plot of stratified analyses of the joint association of sleep disorders and vitamin D deficiency with all-cause, cardiovascular disease and cancer mortality.

# Table S1 The resource and definition of the selected covariates.

| **Covariates** | **Code** | **Description** | **Type** |
| --- | --- | --- | --- |
| **Age** | RIDAGEYR | Age in years of the participant at the time of screening. Individuals 80 and over are topcoded at 80 years of age. | Continuous |
| **Sex** | RIAGENDR | Gender of the participant. | Categorical |
| **Race** | RIDRETH1 | Recode of reported race and Hispanic origin information. | Categorical |
| **Poverty-to-income ratio (PIR)** | INDFMPIR | A ratio of family income to poverty guidelines. | Categorical |
| **Education** | DMDEDUC2  DMDEDUC3 | The highest grade or level of school that participants completed or the highest degree participants received. | Categorical |
| **Health insurance** | HIQ011 | Determined by whether individuals were covered by health insurance or another healthcare plan. | Categorical |
| **Body mass index (BMI)** | BMXBMI | Body Mass Index (kg/m^2^). | Categorical |
| **Smoking status** | SMQ020 | Smoked at least 100 cigarettes in life or not. | Categorical |
| **Alcohol consumption** | ALQ120Q  ALQ121 | Frequency of participant drinking alcohol over past 12 months. | Categorical |
| **Systemic Immune-Inflammation Index**    **Physical activity**  **Hypertension**  **Diabetes**  **Hyperlipidemia**  **Cardiovascular disease**  **Cancer** | LBXPLTSI  LBXNE  LBXLYP  PADLEVEL  BPQ020  BPQ040A  DIQ010  DIQ050  DID070  DIQ070  LBXTC  LBXTR  LBDLDLM  LBDHDD  MCQ160B~  MCQ160F  C00~C97 | Calculated by multiplying the platelet count by the neutrophil count and then dividing by the lymphocyte count.  Measured in MET-minutes.  Hypertension status was determined by whether individuals had been informed by a doctor of high blood pressure or were using antihypertensive medication.  Identified based on any of the following criteria: a self-reported diagnosis of DM by a doctor, glycated hemoglobin A1c (HbA1c) levels ≥6.5%, use of insulin or anti-diabetic medications, fasting glucose levels ≥7.0 mmol/L, random glucose levels ≥11.1 mmol/L, or an oral glucose tolerance test (OGTT) result ≥11.1 mmol/L.  Characterized by any of the following criteria: total cholesterol ≥200 mg/dL, triglycerides ≥150 mg/dL, low-density lipoprotein (LDL) ≥130 mg/dL, or high-density lipoprotein (HDL) <40 mg/dL  Identified based on self-reported physician diagnosis and standardized NHANES health questionnaires.  Defined as individuals who had been informed by a doctor or healthcare professional that they had cancer or any malignancy. | Continuous  Continuous  Categorical  Categorical  Categorical  Categorical  Categorical |

**Table S2** Baseline characteristics of the study participants by categories of sleep disorders.

|  | **Participants stratified by sleep disorders** | | | | |
| --- | --- | --- | --- | --- | --- |
| **Characteristic** | **Total** | **No**  **Sleep Disorders** | **Sleep Disorders** | **P- value** | |
| **Number of subjects** | 24566 | 22499 | 2067 | |  |
| **Sex, n (%)***^c^* |  |  |  | | 0.004 |
| Male | 9987 (48.1) | 9068 (47.8) | 919 (51.5) | |  |
| Female | 10561 (51.9) | 9706 (52.2) | 855 (48.5) | |  |
| **Age, years (mean ± SD)** *^a^* | 49.8 ± 17.8 | 49.4 ± 18.0 | 53.7 ± 15.3 | | <0.001 |
| **Race or ethnicity, n (%)***^c^* |  |  |  | | <0.001 |
| Non-Hispanic White | 9802 (69.6) | 8818 (69.0) | 984 (75.5) | |  |
| Non-Hispanic Black | 4186 (10.8) | 3811 (10.8) | 375 (11.0) | |  |
| Mexican American or Hispanic | 4922 (13.3) | 4596 (13.7) | 326 (9.2) | |  |
| Others | 1638 (6.3) | 1549 (6.5) | 89 (4.2) | |  |
| **PIR, n (%)***^c^* |  |  |  | | <0.001 |
| < 1.30 | 5773 (21.2) | 5194 (20.9) | 579 (24.5) | |  |
| 1.30–3.49 | 7077 (35.2) | 6496 (35.4) | 581 (32.7) | |  |
| ≥ 3.50 | 6133 (43.6) | 5639 (43.7) | 494 (42.8) | |  |
| **Education level, n (%)***^c^* |  |  |  | | 0.001 |
| Less than high school | 9777 (39.1) | 8962 (39.2) | 815 (38.9) | |  |
| High school | 5989 (31.5) | 5417 (31.2) | 572 (34.2) | |  |
| College or higher | 4762 (29.4) | 4375 (29.6) | 387 (26.9) | |  |
| **Health insurance, n (%)***^c^* |  |  |  | | <0.001 |
| No insurance | 4614 (20.1) | 4361 (20.5) | 253 (15.1) | |  |
| Government insurance | 3645 (13.3) | 3145 (12.4) | 500 (22.4) | |  |
| Private insurance | 11107 (66.7) | 10234 (67.1) | 873 (62.5) | |  |
| **Smoking status, n (%)***^c^* |  |  |  | | 0.02 |
| Never smoker | 383 (4.8) | 354 (5.0) | 29 (3.2) | |  |
| Former smoker | 4915 (51.2) | 4377 (50.9) | 538 (53.6) | |  |
| Current smoker | 4052 (44.1) | 3639 (44.2) | 413 (43.2) | |  |
| **Alcohol consumption, n (%)***^c^* |  |  |  | | 0.87 |
| No | 5263 (22.9) | 4796 (22.7) | 467 (24.0) | |  |
| Yes | 13655 (77.1) | 12462 (77.3) | 1193 (76.0) | |  |
| **SII***^b^* | 471.2 (336.8, 665.2) | 470.0 (336.4, 662.9) | 485.5 (343.7, 687.7) | | 0.01 |
| **Physical Activity, MET/week** *^b^* | 1080.0 (80.0, 3600.0) | 1120.0 (120.0, 3600.0) | 615.0 (0.0, 2880.0) | | <0.001 |
| **Vitamin D, nmol/L** *^b^* | 59.9 (43.5, 77.4) | 59.9 (43.4, 77.3) | 60.4 (44.1, 78.6) | | 0.24 |
| **BMI, kg/m²** *^b^* | 27.9 (24.3, 32.4) | 27.6 (24.1, 31.9) | 31.8 (27.2, 37.7) | | <0.001 |
| **History of hypertension, n (%)***^c^* |  |  |  | | <0.001 |
| No | 11458 (61.8) | 10780 (63.7) | 678 (42.2) | |  |
| Yes | 8778 (38.2) | 7695 (36.3) | 1083 (57.8) | |  |
|  | **Participants stratified by sleep disorders** | | | | |
| **Characteristic** | **Total** | **No**  **Sleep Disorders** | **Sleep Disorders** | | **P- value** |
| **History of diabetes mellitus, n (%)***^c^* |  |  |  | | <0.001 |
| No | 17555 (91.2) | 16302 (92.1) | 1253 (80.6) | |  |
| Yes | 2522 (8.8) | 2087 (7.9) | 435 (19.4) | |  |
| **History of hyperlipidemia, n (%)***^c^* |  |  |  | | <0.001 |
| No | 7025 (35.8) | 6621 (36.8) | 404 (25.4) | |  |
| Yes | 13523 (64.2) | 12153 (63.2) | 1370 (74.6) | |  |
| **History of CVD, n (%)***^c^* |  |  |  | | <0.001 |
| No | 18277 (91.4) | 16926 (92.5) | 1351 (80.1) | |  |
| Yes | 2271 (8.6) | 1848 (7.5) | 423 (19.9) | |  |
| **History of cancer, n (%)***^c^* |  |  |  | | <0.001 |
| No | 18541 (90.2) | 17021 (90.8) | 1520 (84.3) | |  |
| Yes | 1989 (9.8) | 1737 (9.2) | 252 (15.7) | |  |

Values are presented as means (standard deviations) for normally distributed continuous variables, medians (interquartile ranges) for non-normally distributed continuous variables, and counts (percentages) for categorical variables.

*^a^* Group comparisons were conducted using Student’s t-test

*^b^* Group comparisons were conducted using the Wilcoxon rank-sum test

*^c^* Group comparisons were conducted using the Chi-square test

PIR, poverty-to-income ratio; BMI, body mass index; CVD, cardiovascular disease; SII, Systemic Immune-Inflammation Index; MET, metabolic equivalent of task; SD: standard deviation.

**Calculation of RERI and ROR**

To calculate additive and multiplicative effects, we conducted an interaction analysis to examine the relationship of between sleep disorders and vitamin D deficiency with mortality, as outlined below:

**Table S3** Interaction test for the relationship of sleep disorder and vitamin D deficiency with mortality.

|  | **All-cause mortality**  **OR (95%CI)** | **CVD mortality**  **OR (95%CI)** | **Cancer mortality**  **OR (95%CI)** |
| --- | --- | --- | --- |
| Multiplicative Interaction | 1.19 (0.85-1.67) | 0.77 (0.38-1.53) | 1.30 (0.64-2.62) |
| Additive interaction |  |  |  |
| RERI | 0.41 (-0.13-1.05) | -0.30 (-1.21-0.89) | 0.61 (-0.55-2.37) |
| AP | 0.20 (-0.11-0.39) | -0.21 (-1.54-0.18) | 0.27 (-0.52-0.51) |
| S | 1.68 (0.84-3.39) | 0.58 (0.07-4.52) | 1.93 (0.52-7.13) |

Adjusted for age, sex, race/ethnicity, educational status, poverty-to-income ratio, health insurance, smoking, alcohol consumption, body mass index, physical activity, Systemic Immune-Inflammation Index and history of cardiovascular disease, diabetes mellitus, hyperlipidemia, hypertension and cancer.

RERI: relative excess risk due to interaction; AP: attributable proportion; S: synergy index; OR: Odds Ratio; CI: Confidence Interval.


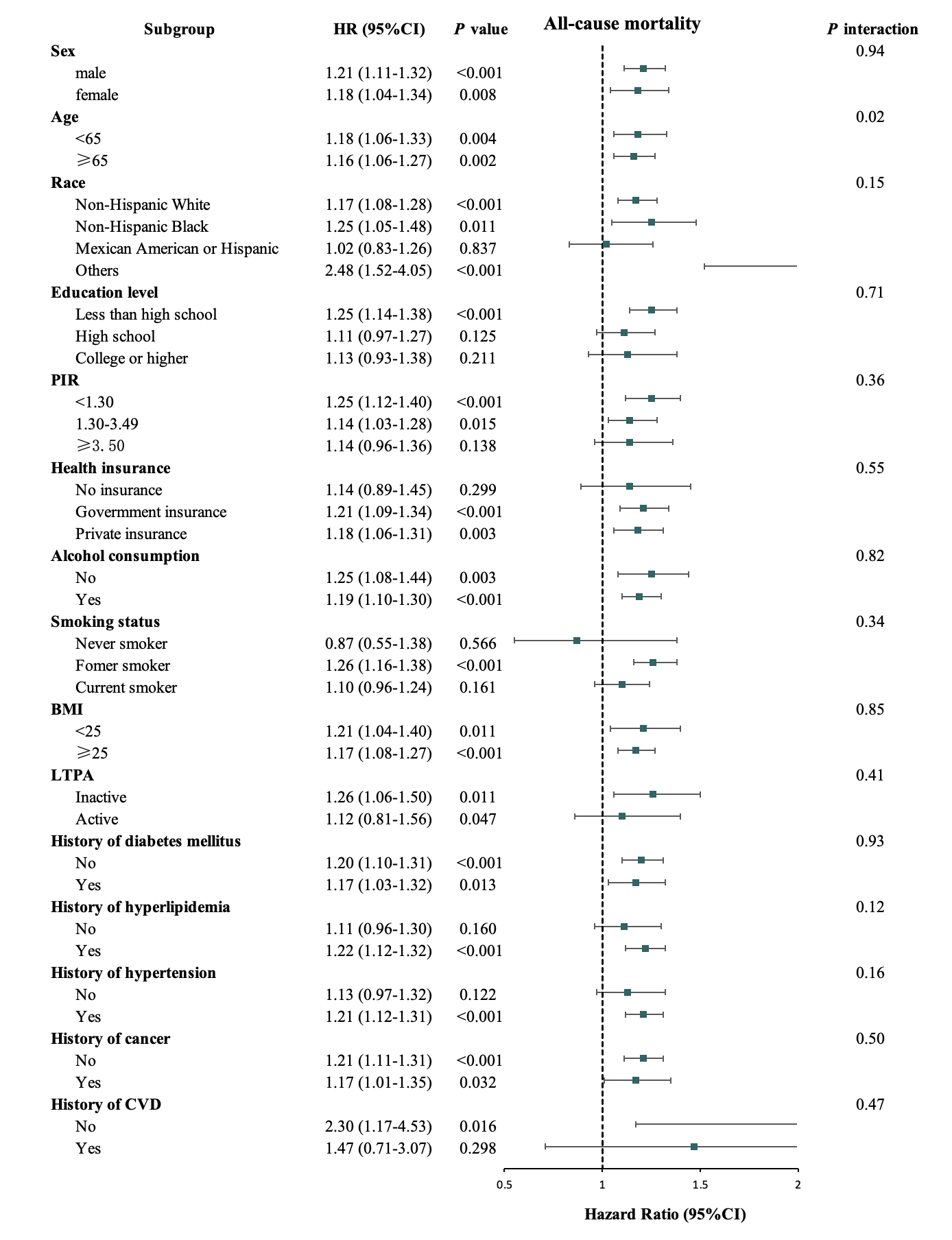


**(A)**


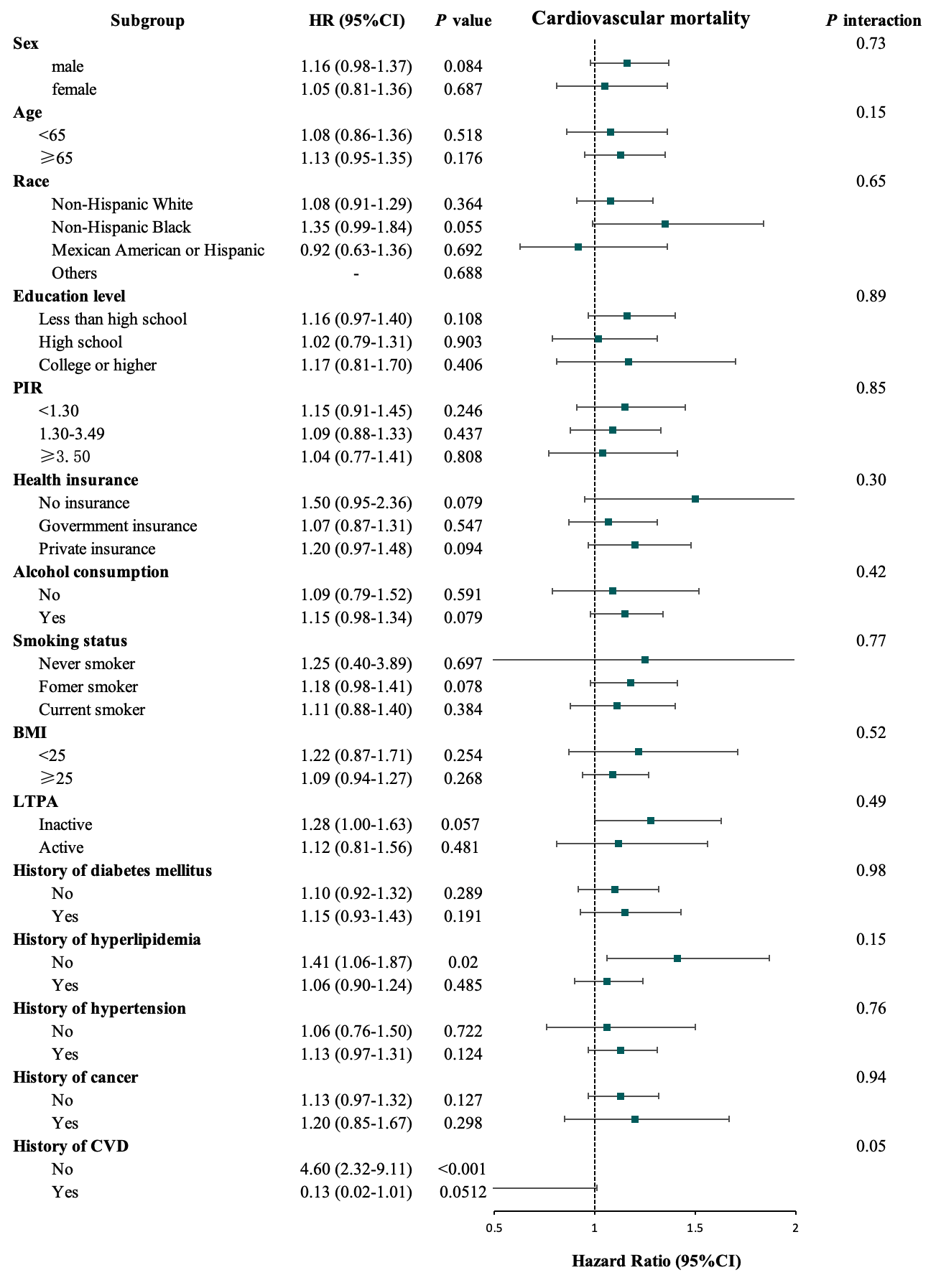


**(B)**


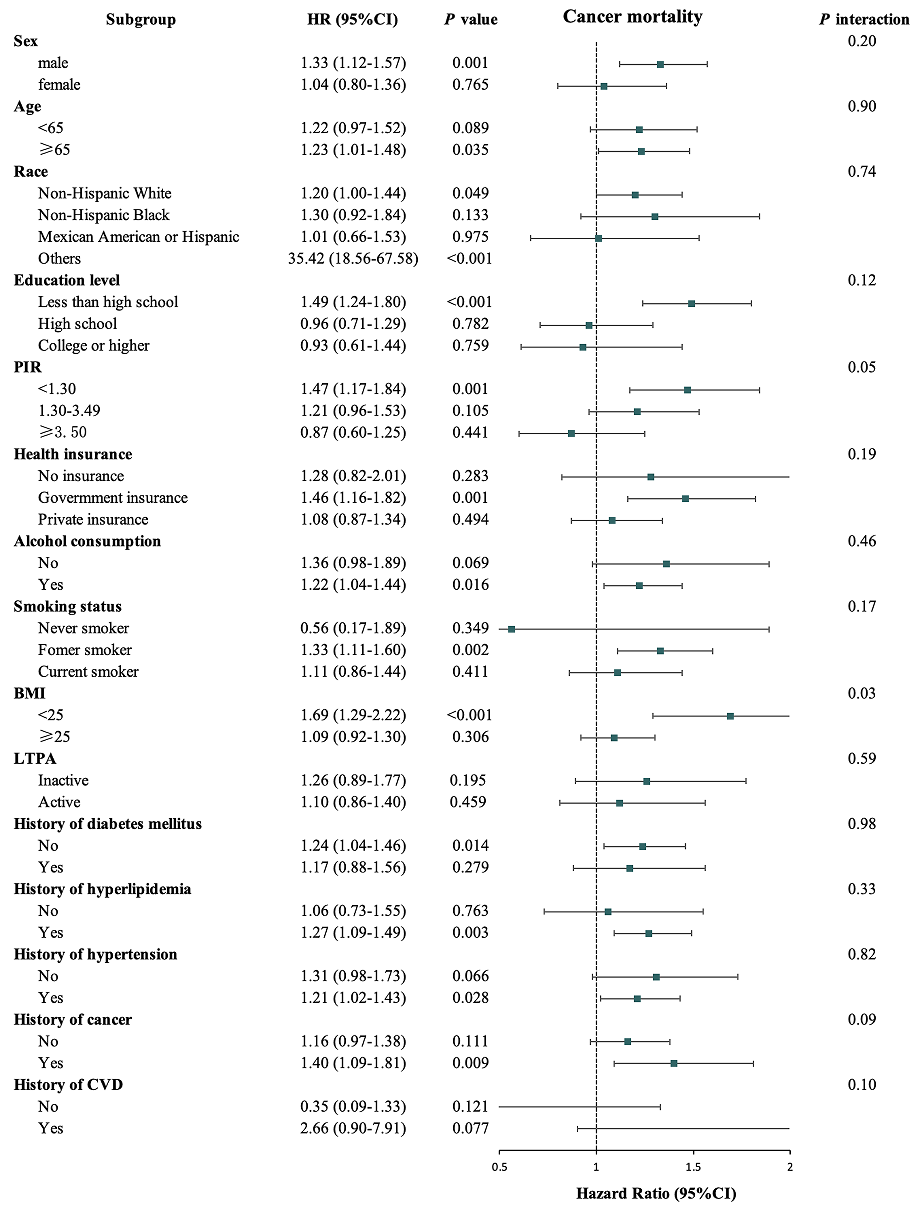


**(C)**

**Fig. S1** Forest plot of stratified analyses of the joint association of sleep disorders and vitamin D deficiency with all-cause, cardiovascular disease and cancer mortality (n = 24566); (A) all-cause mortality; (B) cardiovascular disease mortality; (C) cancer mortality.

Models were adjusted for age, sex, race/ethnicity, educational status, poverty-to-income ratio, health insurance, smoking, alcohol consumption, body mass index, physical activity, Systemic Immune-Inflammation Index, and history of cardiovascular disease, diabetes mellitus, hyperlipidemia, hypertension, and cancer.
